# microRNA-186 in extracellular vesicles from bone marrow mesenchymal stem cells alleviates idiopathic pulmonary fibrosis via interaction with SOX4 and DKK1

**DOI:** 10.1186/s13287-020-02083-x

**Published:** 2021-02-03

**Authors:** Jing Zhou, Yang Lin, Xiuhua Kang, Zhicheng Liu, Wei Zhang, Fei Xu

**Affiliations:** Department of Respiratory and Critical Care Medicine, The First Affiliated Hospital of Nanchang University, No. 17, Yongwaizheng Street, Nanchang, 330006 Jiangxi Province China

**Keywords:** Idiopathic pulmonary fibrosis, Fibroblasts, Human bone marrow mesenchymal stem cells, Extracellular vesicles, microRNA-186, SRY-related HMG box transcription factor 4

## Abstract

**Background:**

Previous reports have identified that human bone marrow mesenchymal stem cell-derived extracellular vesicles (BMSC-EVs) with their cargo microRNAs (miRNAs) are a promising therapeutic approach for the treatment of idiopathic pulmonary fibrosis (IPF). Therefore, we explored whether delivery of microRNA-186 (miR-186), a downregulated miRNA in IPF, by BMSC EVs could interfere with the progression of IPF in a murine model.

**Methods:**

In a co-culture system, we assessed whether BMSC-EVs modulated the activation of fibroblasts. We established a mouse model of PF to evaluate the in vivo therapeutic effects of BMSC-EVs and determined miR-186 expression in BMSC-EVs by polymerase chain reaction. Using a loss-of-function approach, we examined how miR-186 delivered by BMSC-EVs affected fibroblasts. The putative relationship between miR-186 and SRY-related HMG box transcription factor 4 (SOX4) was tested using luciferase assay. Next, we investigated whether EV-miR-186 affected fibroblast activation and PF by targeting SOX4 and its downstream gene, Dickkopf-1 (DKK1).

**Results:**

BMSC-EVs suppressed lung fibroblast activation and delayed IPF progression in mice. miR-186 was downregulated in IPF but enriched in the BMSC-EVs. miR-186 delivered by BMSC-EVs could suppress fibroblast activation. Furthermore, miR-186 reduced the expression of SOX4, a target gene of miR-186, and hence suppressed the expression of DKK1. Finally, EV-delivered miR-186 impaired fibroblast activation and alleviated PF via downregulation of SOX4 and DKK1.

**Conclusion:**

In conclusion, miR-186 delivered by BMSC-EVs suppressed SOX4 and DKK1 expression, thereby blocking fibroblast activation and ameliorating IPF, thus presenting a novel therapeutic target for IPF.

## Background

Idiopathic pulmonary fibrosis (IPF) is a serious progressive lung disease that significantly afflicts 1 million people globally [1]. IPF is a fibrotic interstitial lung disease, characterized by the accumulation of fibroblasts and deposition of collagen, thus damaging lung function [2]. Patients who suffer from IPF have poor prognosis and high morbidity [3], and the current treatment options for IPF are limited [4]. Notably, the proliferation of fibroblasts is a crucial indicator of IPF [5]. Therefore, we attempt in this study attempts to identify a new therapeutic method for IPF based on controlling the functions of fibroblasts.

Mesenchymal stem cells (MSCs) have shown promising therapeutic effects on IPF [6]. In general, MSCs have therapeutic capability for repairing injured tissues by releasing extracellular vesicles (EVs) carrying various active cargoes [7]. In particular, EVs derived from bone mesenchymal stem cell (BMSCs) contain diverse cargoes of proteins, mRNAs, and microRNAs (miRNAs) that impact a range of biological activities in tissue repair [8]. EVs include exosomes (30–150 nm) and microvesicles (100–1500 nm), which together play a vital regulatory role in enabling communication among cells [9]. Previous evidence has identified that BMSC-EVs could attenuate the myofibroblastic differentiation of fibroblasts of IPF and thus alleviate IPF progression [10]. Furthermore, the miRNAs in EVs has been reported to serve as prognostic biomarkers of IPF [11]. miRNAs, a set of small, non-coding RNAs, can modulate gene expression at a posttranscriptional level and which are reportedly involved in the pathologies of PF [12]. Among the miRNAs, miR-186 is poorly expressed in lung tissues of patients with IPF [13]. Interestingly, miR-186 also reduces the collagen V overexpression during IPF, demonstrating its value as a pathogenesis-related biomarker or treatment target [13]. miR-186 has been recently reported to control inflammatory fibroblasts via regulating HIF-1α in chronic obstructive pulmonary disease [14]. Hence, we speculated that BMSC-EVs could deliver miR-186 with beneficial effects on fibroblast activation during IPF.

SOX4 is a transcription factor involved in lung development that regulates the survival of cells [15]. The upregulation of SOX4 has been reported in primary lung tumors [16]. A prior study has demonstrated that the knockdown of SOX4 suppresses the expression of Dickkopf 1 (DKK1), which was otherwise highly expressed in the lung samples from IPF patients [17, 18]. DKK1 is an inhibitor of the Wnt signaling pathway which exerts a key modulatory role in IPF [19, 20]. Our preliminary bioinformatics search predicted that SOX4 could be a target of miR186. Therefore, we attempted to provide experimental support for a new IPF treatment by investigating the influence of BMSC-EVs on fibroblast activation and PF progression in relation to the miR186/SOX4/DKK1 signaling axis.

## Materials and methods

### Ethics statement

Written informed consent was obtained from all patients prior to the study. Study protocols were approved by the Ethics Committee of the First Affiliated Hospital of Nanchang University (No. 201902006) and in accordance with the Ethical Principles for Medical Research involving Human Subjects of the *Declaration of Helsinki*. The animal experiments were performed in accordance with the recommendations in the *Guide for the Care and Use of Laboratory Animals*, and the protocol was approved by the Institutional Animal Care and Use Committee of the First Affiliated Hospital of Nanchang University (No. 201907003).

### Collection of lung tissue samples from patients with IPF

The IPF patients (*n* = 48) from the Pathology Department of the First Affiliated Hospital of Nanchang University that conformed to the statement for diagnosis and treatment of IPF in 2011 were enrolled in this study. The inclusion criteria of the included IPF patients were as follows: the patients diagnosed according to the IPF diagnostic criteria formulated by the Chinese Thoracic Society/American Thoracic Society and the patients presented with typical symptoms such as cough and progressive dyspnea. Meanwhile, patients with the following conditions were excluded: acute and chronic pulmonary infections, sarcoidosis, neoplastic diseases (including solid tumors/non-solid tumors), allergic alveolitis, granulomatous diseases, other types of interstitial lung diseases, or autoimmune diseases; contraindications to electronic bronchoscopy; and patients treated with medium- and high-dose of glucocorticoids (more than 50 mg prednisone/day) within 1 month. The 48 enrolled IPF patients had a mean DL_CO_ of 56.33 ± 18.23 (% predicted) and FVCa of 64.01 ± 16.34 (% predicted). The study population included 26 males and 22 females, with a mean age of 54.13 ± 3.62 years and a mean body mass index (BMI) of 27.55 ± 4.2 kg/m^3^. Twenty-five patients were smokers among the population. The human lung tissue specimens were preserved in liquid nitrogen. Besides, transplanted lung tissue samples obtained from the 16 organ donors (10 males and 6 females; with a mean age of 49.56 ± 3.58 years) were set as the control. The specimens in the control group had not met the necessary standards for lung transplantation.

### Bioinformatics analysis

Microarray dataset GSE35145 obtained from the Gene Expression Omnibus (GEO) database was analyzed using the R language “limma” package (http://www.bioconductor.org/packages/release/bioc/html/limma.html) to obtain the significant differentially expressed genes with the threshold of ∣ logFoldChange∣ > 1 and *p* < 0.05. There were eight samples in the microarray dataset GSE35145, of which four were normal samples and four were PF samples. The downstream genes of miR-186 were predicted by starBase (clipExpNum ≧ 5, pancancerNum > 10). The human transcription factors obtained from Cistrome were intersected with differentially expressed genes and miRNA downstream genes using Venn mapping to obtain the key transcription factors. The downstream genes of key transcription factors were screened based on literature. The relationship between the transcription factor and its downstream genes was demonstrated by MEM and hTFtarget. Also, the genes related to the downstream genes were predicted by String and the protein-protein interaction (PPI) network was then constructed by Cytoscape (https://cytoscape.org). Kyoto Encyclopedia of Genes and Genomes (KEGG) pathway enrichment analysis was performed on these genes through KO-Based Annotation System (KOBAS), and the KEGG pathway maps were drawn using R language.

### BMSC collection and identification

The marrow specimens were provided from three hospitalized patients with femoral head necrosis (two males; one female; aged 26–52 years) in the First Affiliated Hospital of Nanchang University. The diagnosis was based on magnetic resonance imaging (MRI) examination. Patients who had not lost height of the femoral head were recruited as marrow donors, whereas patients with diseases such as trauma, hematological system disorders, or tumor infiltration were excluded. BMSCs were isolated from bone marrow and cultured in Dulbecco’s Modified Eagle Medium/Nutrient Mixture F-12 (DMEM-F12) basal medium (Hyclone, Logan, Utah) containing 10% fetal bovine serum (FBS) (Exosome-depleted; Cat. No.: A2720801; Gibco, NY) and 0.2% penicillin-streptomycin (Hyclone, Logan, Utah). The cells were subcultured every 3 days, and the MSCs at 3rd to 7th passage were used for subsequent experiments. Then, MSCs were cultured in the OriCell™ MSC osteogenic, adipogenic, or chondrogenic differentiation mediums (all purchased from Cyagen, Guangzhou, China). The differentiated BMSCs were identified by Alizarin Red staining, Oil red O staining, and Alcian Blue staining.

### Culture and transfection of fibroblasts

Lung fibroblasts LL29 (from pulmonary tissues of female patient with IPF) were purchased from the American Type Culture Collection (ATCC, Manassas, VA) and incubated with Kaighn’s Modification of Ham’s F-12 (F12K) Medium containing 10% FBS. In total, 100 pmoL mimic-NC, miR-186 mimic, inhibitor-NC, or miR-186 inhibitor (Auragene Bioscience, Changsha, China) diluted in 250 μL serum-free medium was incubated with 10 μL Lipofectamine® 2000 (diluted in 250 μL serum-free medium) for 4 h at 37 °C. A complete medium was utilized for an additional 48-h incubation. Finally, the transfected lung fibroblasts LL29 or BMSCs were collected for subsequent experiments. The plasmids of short hairpin RNA negative control (sh-NC), sh-SOX4, overexpression vector expressing NC (oe-NC), and oe-DKK1 were purchased from Qiagen (Tuttlingen, Germany). Lung fibroblasts LL29 were transfected with the aforementioned plasmids using Lipofectamine RNAiMAX Transfection Reagent (Thermo Fisher Scientific, Waltham, MA) following the manufacturer’s instructions.

### Isolation of EVs

The conditioned medium for BMSCs was harvested every 2–3 days and subsequently preserved at − 80 °C. The cells were centrifuged at 300×*g* for 30 min. Then, the particles (500–1000 nm) were spun at 10,000×*g* for 20 min and allowed to settle, whereas the EVs (50–500 nm) were obtained by another spin at 100000×*g* for 20 min. Next, the isolated EVs were washed with 25 ml PBS and re-spun at 100000×*g* for 1 h and the supernatant was discarded. The final EVs were resuspended by 400 μL PBS for immediate use or preserved at − 80 °C. Finally, the protein concentration of EVs was measured using a bicinchoninic acid (BCA) protein analysis kit (Thermo Fisher, Waltham, MA).

### Transmission electron microscope (TEM)

The suspension of the separated EVs particles was dropped on a carbon-coated copper grid and fixed with a sodium carbonate buffer (pH 7.3 and 0.1 mol/L) supplemented with 2% glutaraldehyde + 2% paraformaldehyde for 3 h at room temperature. The samples were dried at the critical point, fixed on a sample post, followed by carbon sputtering. Finally, the specimen was observed under TEM (Tecnai G2 Spirit BioTWIN, FEI Company, Hillsborough, Oregon) furnished with an Eagle 4 K HS digital camera (FEI).

### Nanoparticle tracking analysis (NTA)

Nanosight NS-300 (Malvern, Worcestershire, UK) tracking instrument was fitted with a 405 nm laser. The exposure time was adjusted to eliminate background noise. Three independent videos at 60-s intervals were recorded using NTA software (Nanosight 2.1).

### EV labeling and immunofluorescence assay (IFA)

EVs were re-suspended in 400 μL PBS at a concentration of 0.1–0.2 μg. The EVs were dyed by CellMask Deep Red (Thermo Fisher Scientific), with excitation/emission wavelength at 649/666 nm. EVs were stained with crimson dye (1:1000) at 37 °C for 20 min for labeling and washed with PBS (1 to 10,000 v/v ratio) to remove the unbound dye. Then, the EVs were separated by centrifugation at 100,000×*g* for 1 h and resuspended in PBS. Next, the EVs were stained with CellTrace™ carboxyfluorescein succinimidyl ester (CFSE, Life Technologies, Carlsbad, CA) and observed at 492/517 nm. The CFSE dyes were permeated into detached cells using endoesterase and subsequently reacted with intracellular primary amines to form a stable and non-diffusible fluorescent staining. LL29 cells (3–5 × 10^5^) in serum-free medium were dyed by CFSE at a 1:1000 dilution (5 μM) and incubated at 37 °C for 20 min under conditions devoid of light. The solution was then centrifuged and the pellet was rinsed with a serum-free medium at a ratio of 1:10 to eliminate the unbound dye.

EVs were then cultured in an 8-well chambered slide (Millipore, Burlington, MA). CFSE-dyed cells were treated with EVs, fixed with 3.7% (w/v) formaldehyde at ambient temperature for 5 min, and subsequently imaged with a fluorescence microscope.

### Reverse transcription-quantitative polymerase chain reaction (RT-qPCR)

After RNA extraction using TRIzol kit (Invitrogen, Grand Island, NY), the complementary DNA (cDNA) was synthesized by a reverse transcription kit (Takara, Dalian, Liaoning, China) using random primers. mRNA levels of α-SMA, collagen I, and SOX4 were quantitatively detected by the SYBR Green kit (Exiqon, Denmark). For detection of the expression of miR-186, real-time qPCR were performed using the Bulge-LoopTM qPCR kit (RiboBio, Guangzhou, China) according to the manufacturer’s protocol. β-actin was set as an internal reference for mRNA expression, whereas U6 was set as an internal reference for miRNA expression. The commercial miR-186 primer sets (from RiboBio, Guangzhou, China) and mRNA primers (Table 1) were designed and synthesized by Sangon Biotech Co., Ltd. (Wuhan, Hubei, China). The relative expression of the transcript was quantified with 2^−ΔΔCT^ method. The experiment was repeated three times in triplicate.

**Table 1:** Primer sequences for RT-qPCR

Target	Primer sequences
α-SMA (hsa)	F 5-TATCCCCGGGACTAAGACGGG-3
	R 5-CAGAGCCCAGAGCCATTGTC-3
α-SMA (mmu)	F 5-CGAGCGTGAGATTGTCCGT-3
	R 5-CCCCTGACAGGACGTTGTT-3
Collagen I (hsa)	F 5-CGAAGACATCCCACCAATCAC-3
	R 5-CAGATCACGTCATCGCACAAC-3
Collagen I (mmu)	F 5-ACGCATGGCCAAGAAGACAT-3
	R 5-TTGTGGCAGATACAGATCAAGCA-3
SOX4 (hsa)	F 5-AACCCCAGCTCAAACTTTGAGA-3
	R 5-GAACCCCAGCTCAAACTTTGAG-3
SOX4 (mmu)	F 5-AACCCCAGCTCAAACTTTGAGA-3
	R 5-GAACCCCAGCTCAAACTTTGAG-3
DKK1 (hsa)	F 5-CGCCGAAAACGCTGCAT-3
	R 5-TTTCCTCAATTTCTCCTCGGAA-3
DKK1 (mmu)	F 5-GGGAGTTCTCTATGAGGGCG-3
	R 5-AAGGGTAGGGCTGGTAGTTG-3
U6 (hsa and mmu)	F 5-AGGCGTCACTTTGACTTGG-3
β-actin (hsa)	F 5-AGG GGCCGGACTCGTCATACT-3
	R 5-GGCGGCAC CACCATGTACCCT-3
β-actin (mmu)	F 5-GTGACGTTGACATCCGTAAAGA-3
	R 5-GCCGGACTCATCGTACTCC-3
miR-186	F 5-GGGCAAAGAATTCTCCTTT-3
	R 5-GTGCAGGGTCCGAGGT-3

*α-SMA* alpha-smooth muscle actin, *SOX4* SRY-related HMG box transcription factor 4, *DKK1* Dickkopf-1, *miR-186* microRNA-186

### Western blot analysis

The total protein extracted from the cell lysate using RIPA buffer (Beyotime, Shanghai, China) was quantified by a BCA assay kit (Beyotime). After 10% sodium dodecyl sulfate-polyacrylamide gel electrophoresis (SDS-PAGE), the separated proteins were electrotransferred onto the polyvinylidene fluoride (PVDF) membrane (Millipore, Boston, MA). Subsequently, the membrane was blocked with 5% skimmed milk and probed with the following primary antibodies from Abcam (Cambridge, UK) rabbit anti-α-SMA (ab32575, 1:2500), rabbit anti-collagen I (ab34710, 1:2500), rabbit anti-SOX4 (ab80261, 1:1500), rabbit anti-DKK1 (ab93017, 1:1500), rabbit anti-β-actin (ab8226, 1:5000), rabbit anti-CD63 (ab134045, 1:1000), rabbit anti-CD81 (ab109201, 1:5000), rabbit anti-CD9 (ab92726, 1:1000), rabbit anti-Alix (ab88388, 1:1000), rabbit anti-TSG101 (ab125011, 1:1000), and rabbit anti-calnexin (ab92573, 1:20000) at 4 °C in the dark. Subsequently, the membrane was re-probed with horseradish peroxidase (HRP)-combined immunoglobulin G (IgG) antibodies (ab6721, 1:5000, Abcam) for 2 h followed by enhanced chemiluminescence (BB-3501, Ameshame, UK) for immunodetection. The protein bands were imaged and analyzed by the BIO-RAD imaging system (California).

### Cell Counting Kit-8 (CCK-8) assay

The transfected cells or cells treated with EVs for 24 h were cultured overnight in a 96-well plate, 2.5 × 10^4^ cells/well. The Cell Counting Kit-8 (Dojindo Molecular Technologies, Kyushu, Japan) was utilized to detect cell viability. In brief, each petri dish was loaded with 10 μL CCK8 solution and subsequently incubated at 37 °C for 2 h, and the cell viability was measured using Wellscan MK3 (Labsystems Dragon, Finland) employing a wavelength of 450 nm. The experiment was repeated three times in triplicate.

### Transwell assay

The fibroblasts were starved for 24 h, and the cells were then adjusted to the final cell concentration of 2 × 10^5^/mL. 0.2 mL cell suspension was loaded onto the upper Transwell chamber, whereas the lower one was loaded with 700 μL pre-chilled F12K medium containing 10% FBS. Two chambers were placed in an incubator of 5% CO_2_ at 37 °C. After 24 h of incubation, the cells attached on the upper chamber and basement membrane were wiped off followed by fixation with methanol for 30 min. After crystal violet staining (0.1%) for 20 min, the cells were subsequently pictured under an inverted microscope (CKX41, Olympus Corporation, Tokyo, Japan). The trans-membrane cells in five randomly selected fields were counted.

The extracellular matrix (ECM) gel was placed at 4 °C overnight. On the next day, after all the pipette tips and chambers had been pre-chilled on ice for 30 min, the fibroblasts were diluted with the serum-free medium (1:9) to achieve a final concentration of 1 mg/mL. The polycarbonate membrane in each Transwell upper chamber (24 wells) was cultured with 40 μL of ECM gel and incubated in a cell incubator with 5% CO_2_ at 37 °C for 5 h to allow polymerization of ECM gel. After removing the excess liquid, the fibroblasts were added with 70 μL of F12K medium and incubated in a 37 °C incubator for half an hour to rehydrate the Matrigel for subsequent experiments. After starving for 24 h, serum was removed and the cells were re-suspended in F12K medium without FBS to achieve a final cell concentration of 2.5 × 10^5^/mL. The hydrated basement membrane in the upper chamber was added with 0.2 mL suspension and the lower chamber was added with 700 μL pre-chilled F12K medium containing 10% FBS. These chambers were incubated in a saturated humidity incubator with 5% CO_2_ at 37 °C for 24 h. The following steps were the same with the Transwell migration assay described above.

### Cell cycle detection

Fibroblasts in logarithmic growth phase were starved for 24 h. After 96 h, the cells were rinsed with PBS, fixed with 70% ethanol, and preserved at − 20 °C for at least 24 h. Following centrifugation at 500*g* for 5 min, cells were suspended in 500 μL of PI/RNase staining buffer and incubated at room temperature in dark for 30 min. Cell cycle was analyzed using BD FACSVerse (BD Biosciences, San Jose, CA). Data were analyzed using FlowJo software.

### Apoptosis detection

The fibroblasts were collected and centrifuged with the supernatant discarded. Cells were rinsed three times with precooled PBS and centrifuged to remove the supernatant. Following the instructions of AnnexinV-fluorescein isothiocyanate (FITC) apoptosis detection kit (K201-100, Biovision Inc., CA), the AnnexinV-FITC/propidium iodide (PI) staining solution was prepared. Specifically, AnnexinV-FITC, PI, and 4-(2-hydroxyethyl)-1-piperazine ethane sulfonic acid (HEPES) buffer were mixed at a ratio of 1:2:50. 1 × 10^6^ cells were resuspended in each 100 μL volume of AnnexinV-FITC/PI staining solution and incubated with 1 mL HEPES buffer (PB180325, Wuhan Procell Life Technology Co., Ltd.) for 15 min at room temperature. The cells were excited at 488 nm, and FITC fluorescence was detected using a 525-nm band-pass filter while PI fluorescence detected with a 620-nm band-pass filter.

### Dual-luciferase reporter gene assay

The binding relation of SOX4 and miR-186 was predicted by StarBase. The potential binding fragment of miR-186 in SOX4 3′UTR and the fragment of mutant SOX4 3′UTR were cloned into individual pGLO vectors, designated as pGLO-SOX4-wild type (WT) and pGLO-SOX4-mutant type (MUT) plasmids, respectively. HEK-293 T cells (Invitrogen) were then co-transfected with miR-186 mimic or mimic NC and the above-mentioned reporter plasmids for 24 h, and the supernatant was collected. The relative luciferase activity (firefly luciferase/Renilla luciferase) was obtained using the dual-luciferase reporter gene detection system (E1910, Promega, Madison, WI).

### Establishment of the pulmonary fibrosis mice model

The C57BL/6 male mice (6–10 weeks, 16–21 g) were subcutaneously injected with 80 mL of ketamine (3.2 mg/kg) and xylazine (0.16 mg/kg) for anesthesia. The trachea was exposed with a midline incision on the neck skin, and then 0.2 mL of bleomycin (5 mg/kg, Sigma, San Francisco, CA) dissolved in saline was evenly delivered into the trachea. The sham-operated mice were infused with normal saline. Seven days after the PF modeling was initiated, a total of 100 μg BMSC-EVs (EVs derived from BMSCs transfected with miR-186 inhibitor or miR inhibitor NC) in 0.2 mL PBS was injected into the mice via the tail vein, with PBS as the control. The mice were intraperitoneally injected with overdose ketamine (5 mg/kg) and xylazine (100 mg/kg) 21 days after PF modeling to collect the lung tissues. Next, half portion was fixed in formaldehyde for dehydration followed by paraffin embedding and further staining, whereas the other half was stored in liquid nitrogen for analysis.

### Histological staining

The lung tissues of successfully established modeled mice were fixed in 10% formalin buffer and paraffin-embedded after dehydration in an ethanol series. Then, the tissues were cut into 5-μm-thick sections, which were stained with hematoxylin-eosin (HE). The sections were stained with hematoxylin for 10 min, and then stained with aniline blue solution for 5 min, followed by staining with 1% acetic acid solution for 2 min. Subsequently, the morphology of connective tissues was observed after performing Masson’s trichrome staining. Meanwhile, the collagen expression in the lung tissues was detected by Sirius Red staining by immersion in 1% Sirius Red/saturated picric acid solution for 1 h. Finally, the tissues were washed with 0.5% acetic acid prior to slide mounting.

### Immunofluorescence cell-counting assay for fibroblasts

Immunofluorescence staining was performed to assess the fluorescence intensity of fibroblast specific marker TE-7 in the lung tissue sections of mice treated with BMSC-EVs so as to evaluate the effects of BMSC-EVs on the number of fibroblasts [21, 22]. Formalin-fixed and paraffin-embedded lung sections were fixed with 4% paraformaldehyde, soaked in 0.3% Triton X-100 in PBS, and incubated with TE-7 antibody (CBL271, 1:100; Chemicon, Billerica, MA). Alexa-Fluor conjugated antibody served as secondary antibody. The nuclei were stained with DAPI (4′,6-diamino-2-phenylindole dihydrochloride). The fluorescence intensity was observed using a LSM 710 microscope (Carl Zeiss, Oberkochen, Germany).

### Hydroxyproline assay

The degree of IPF was assessed by detecting collagen content in mouse lung tissues using conventional hydroxyproline method. The lung tissue was dried under vacuum and hydrolyzed with 6N hydrochloric acid overnight at 120 °C. The hydroxyproline content (μg/mg) was determined in the right lung of mice. The capacity to hydrolyze and recover hydroxyproline from collagen was confirmed with reference to purified collagen samples.

### Statistical analysis

All data were analyzed by SPSS 21.0 software (IBM, Armonk, NY), with *p* < 0.05 as the level of significance. Measurement data were presented as the mean ± standard deviation. Differences between two groups were compared by unpaired *t* test, while differences among groups were determined by one-way analysis of variance (ANOVA), followed by Tukey’s post hoc test. The comparisons at different time points were performed using two-way ANOVA followed by Bonferroni’s post hoc test. Pearson’s correlation coefficient was employed to analyze correlations.

## Results

### BMSC-EVs suppress the lung fibroblast activation

**Fig. 1:**
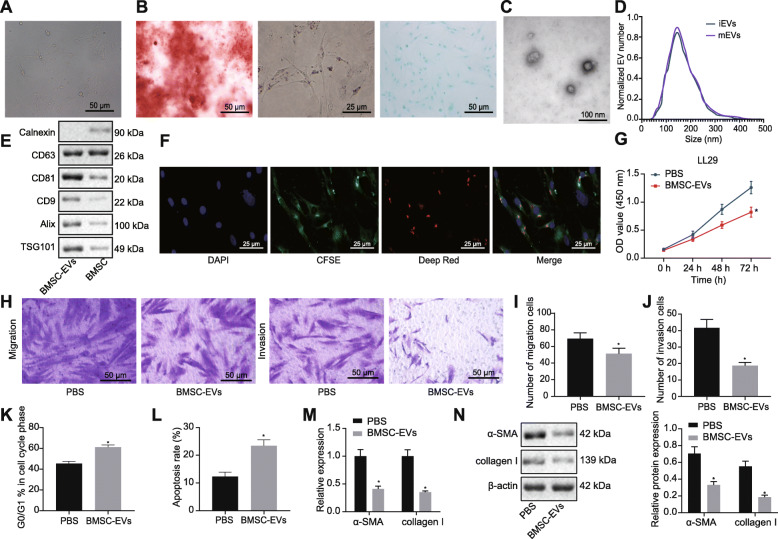
BMSC-EVs suppresses the activation of lung fibroblasts. **a** The morphology of human-derived BMSCs observed under a light microscope (× 200). **b** Representative images of osteocytes (× 200), adipocytes (× 400), and chondrocytes (× 200) differentiated from BMSCs using differentiation medium, shown by Alizarin Red staining (I), Oil Red O staining (II), and Alcian Blue staining (III). **c** BMSC-EVs observed by an electron microscope. **d** The distribution of EVs in size determined by NTA. iEVs, infectious extracellular vesicles; mEVs, mock-infected extracellular vesicles. **e** The protein expression of CD63, CD81, CD9, Alix, TSG101, and calnexin in BMSC-EVs and BMSC lysate determined by Western blot analysis. **f** The immunofluorescence image of BMSC-EVs internalized by human lung fibroblasts at the 24th hour (dual labels; red-exosomes-50%; green-lung fibroblasts; 630 times). **g** The proliferation of the fibroblasts assessed by CCK8 assay after treatment with BMSC-EVs. **h**, **i** The migration of the fibroblasts after treatment with BMSC-EVs evaluated by Transwell assay. **j** The invasion of the fibroblasts after treatment with BMSC-EVs evaluated by Transwell assay. **k** Cell cycle of the fibroblasts after treatment with BMSC-EVs analyzed by flow cytometry. **l** Apoptosis of the fibroblasts after treatment with BMSC-EVs determined by flow cytometry. **m** The mRNA expression of myofibroblast markers, α-SMA, and collagen I in the fibroblasts after treatment with BMSC-EVs determined by RT-qPCR. **n** The protein expression of α-SMA and collagen I in the fibroblasts after treatment with BMSC-EVs measured by western blot analysis. *p < 0.05 vs. the fibroblasts treated with PBS. Differences between the two groups were compared by unpaired *t* test

BMSC-EVs can be internalized by lung fibroblasts to impede the myoblast differentiation of the lung fibroblasts [10]. Hence, in this study, we isolated and identified BMSCs (Fig. 1a, b), and then isolated the BMSC-derived EVs. BMSC-EVs had an average diameter of 155 ± 2.8 nm and expressed the CD63, CD81, CD9, Alix, and TSG101 protein markers, but did not express calnexin protein (Fig. 1c–e). Next, fibroblasts (LL29 cells) were stained with CFSE, whereas the BMSC-EVs were pre-stained with a deep-red lipophilic fluorescent dye, and then 10 μg portions of the aforementioned BMSC-EVs were co-cultured with CFSE-dyed fibroblasts for 24 h. As shown in Fig. 1f, internalization/uptake of BMSC-EVs by fibroblasts was detected by fluorescence microscopy.

The 10 μg samples of BMSC-EVs were utilized to treat the fibroblasts for 24 h, and subsequently, the viability and invasion of fibroblasts were detected. Results of CCK8 and Transwell assay demonstrated that the viability and invasion of the fibroblasts were significantly diminished by BMSC-EVs (Fig. 1g–j). Additionally, the proportion of fibroblasts at G0/G1 phase and apoptosis were significantly increased after treatment with BMSC-EVs (Fig. 1k, l). RT-qPCR and Western blot analyses identified significantly reduced expression of α-SMA and collagen I in the fibroblasts in response to treatment with BMSC-EVs (Fig. 1m, n).

### BMSC-EVs suppress IPF in vivo

**Fig. 2:**
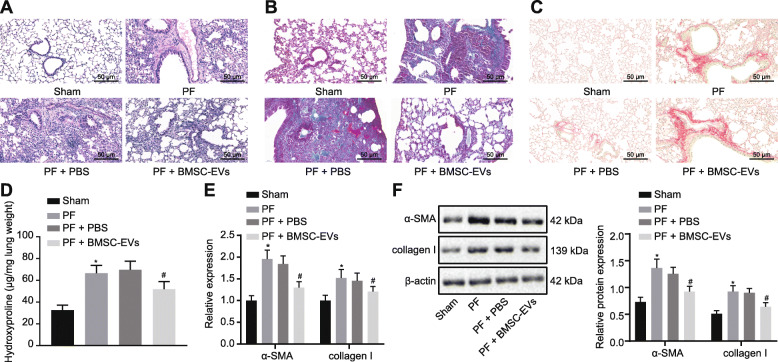
BMSC-EVs suppresses IPF in vivo. **a**–**c** HE staining (**a**), Masson staining (**b**), and Sirius Red staining (**c**) images of the lung tissues of mice with PF (× 200). **d** Hydroxyproline content in lung tissues of mice with PF. **e**, **f** The mRNA (**e**) and protein (**f**) expression of myofibroblastic markers, α-SMA, and collagen I in lung tissue of mice with PF measured by RT-qPCR and Western blot analysis. *p < 0.05 vs. PF mice treated with PBS; ^#^*p* < 0.05 vs. PF mice treated with BMSC-EVs. Differences among groups were analyzed by one-way ANOVA (*n* = 8)

To test the potential therapeutic function of BMSC-EVs, PF mouse model was induced. HE staining (Fig. 2a) identified significant fibrosis in the lung tissues of the PF mice, which was partially alleviated by BMSC-EV treatment. Masson staining (Fig. 2b) demonstrated the presence of collagen fibers in untreated PF mice, whereas collagen expression was reduced by BMSC-EV treatment. Results from Sirius Red staining also revealed the upregulation of collagen fibers in PF mice, and the partial alleviation of this pathology by BMSC-EV treatment (Fig. 2c). As shown in Fig. 2d, the hydroxyproline content was higher in the lung tissue of PF mice compared to the sham-operated mice, whereas this high hydroxyproline content was decreased by BMSC-EVs treatment. Next, the determination of myofibroblastic markers demonstrated that the treatment with BMSC-EVs resulted in reduced expression of myofibroblastic markers, α-SMA, and collagen I (Fig. 2e, f).

### miR-186 in BMSC-EVs impairs the fibroblast activation

**Fig. 3:**
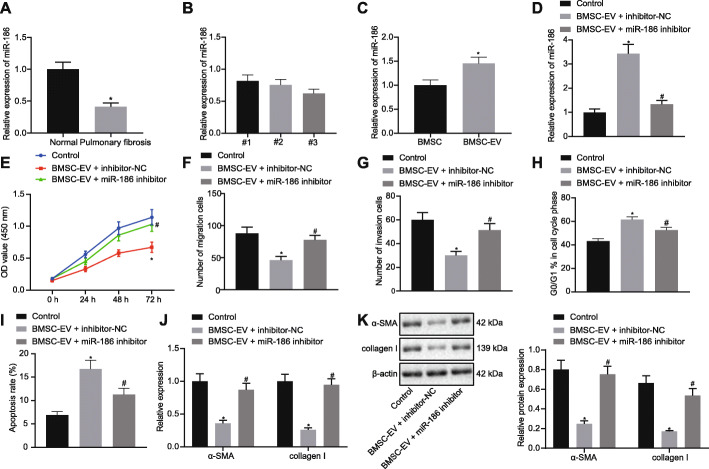
miR-186 in BMSC-EVs inhibits the fibroblast activation. **a** The expression of miR-186 in lung tissues of patients with IPF (*n* = 48) and control donors (*n* = 16) determined by RT-qPCR. **b** Expression of miR-186 in the EVs secreted by BMSCs from three hospitalized patients with femoral head necrosis determined by RT-qPCR. **c** The miR-186 expression in BMSCs and BMSC-EVs determined by RT-qPCR. In **d**–**k**, fibroblasts LL29 were treated with PBS or EVs from BMSCs transfected with miR-186 inhibitor or inhibitor-NC. **d** The detection of miR-186 expression in the fibroblasts by RT-qPCR. **e** The proliferation of fibroblasts assessed by CCK8. **f**, **g** The migration and invasion of the fibroblasts evaluated by Transwell assay. **h** Cell cycle of fibroblasts analyzed by flow cytometry. **i** Apoptosis of fibroblasts evaluated by flow cytometry. **j**, **k** The mRNA (**j**) and protein (**k**) expression of myofibroblastic markers α-SMA and collagen I in the fibroblasts measured by RT-qPCR and western blot analysis. *p < 0.05 vs. the control donors, case#1, BMSCs or fibroblasts treated with PBS; ^#^*p* < 0.05 vs. the fibroblasts treated with EVs from BMSCs transfected with inhibitor-NC. Differences between two groups were compared by unpaired *t* test, while differences among groups were determined by one-way ANOVA, followed by Tukey’s multiple comparisons post-test. Besides, the comparison of the data in each group at different time points was analyzed by two-way ANOVA. The experiment was repeated three times

Previous studies have shown that miR-186 plays an important regulatory role in PF [13, 23]. Therefore, we investigated whether the activation of the fibroblasts was induced by miR-186 delivered via BMSC-EVs. Here, we detected the expression of miR-186 in lung tissues of patients with IPF and control donors. As shown in Fig. 3a, the expression of miR-186 was significantly downregulated in lung tissues of PF patients as compared to the control donors. Then, we detected the expression of miR-186 in the EVs secreted by BMSCs from three hospitalized patients with femoral head necrosis. A highest expression level of miR-186 was witnessed in the BMSC-EVs isolated from case #1 (Fig. 3b). Consequently, we performed subsequent experiments using the BMSCs isolated from case #1. Meanwhile, the expression of miR-186 was higher in BMSC-EVs relative to BMSCs (Fig. 3c). To explore the effect of miR-186 in BMSC-EVs on fibroblasts, transfection of inhibitor-NC/miR-186 inhibitor into BMSCs was conducted before EV isolation, and the isolated BMSC-EVs or PBS was added into the fibroblasts. Results demonstrated that miR-186 expression was dramatically elevated in the fibroblasts treated with EVs from BMSCs transfected with inhibitor-NC relative to the fibroblasts treated with PBS. However, miR-186 expression was notably diminished in the fibroblasts treated with EVs from BMSCs transfected with miR-186 inhibitor as compared to the fibroblasts treated with EVs from BMSCs transfected with inhibitor-NC (Fig. 3d). CCK8 and Transwell assays demonstrated that the proliferation, migration, and invasion of the fibroblasts were notably decreased by treatment with EVs from BMSCs transfected with inhibitor-NC, but these properties were significantly enhanced when miR-186 expression from BMSC-EVs was inhibited (Fig. 3e–g). Furthermore, the proportion of fibroblasts arrested at G0/G1 phase was increased and apoptosis was enhanced by treatment with EVs from BMSCs transfected with inhibitor-NC, whereas these changes were neutralized when miR-186 expression from BMSC-EVs was inhibited (Fig. 3h, i). In addition, expression of α-SMA and collagen I was significantly reduced in the fibroblasts treated with EVs from BMSCs transfected with inhibitor-NC as compared to those treated with PBS, whereas significant higher expression of α-SMA and collagen I was detected in the fibroblasts when miR-186 in BMSC-EVs was inhibited (Fig. 3j, k).

### miR-186 in EVs targets SOX4 in the fibroblasts

**Fig. 4:**
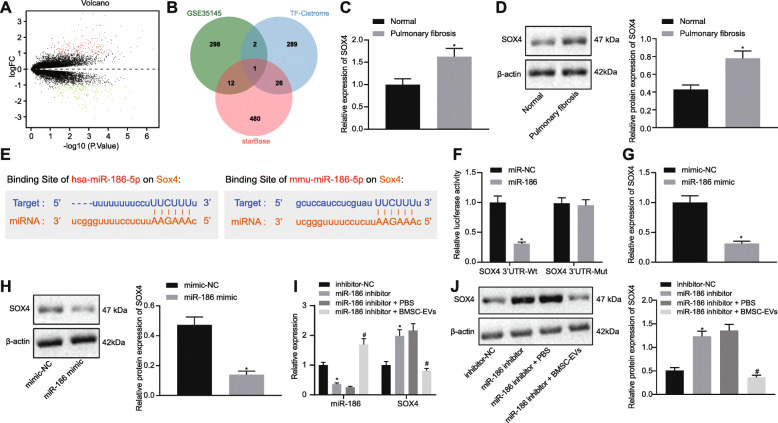
miR-186 in EVs directly targets SOX4 in the fibroblasts. **a** The volcanic map of differentially expressed genes in PF obtained by differential analysis of microarray GSE35145 (red: the upregulated genes; green: the downregulated genes) obtained from GEO database (https://www.ncbi.nlm.nih.gov/gds). **b** Venn maps of differentially expressed genes obtained from microarray GSE35145, miR-186 downstream genes predicted by StarBase (http://starbase.sysu.edu.cn/), and human transcription factors in Cistrome (http://cistrome.org) (the intersection gene: SOX4). **c**, **d** SOX4 expression in clinical lung tissues of patients with IPF (*n* = 48) and control donors (*n* = 16) measured by RT-qPCR and Western blot analysis. **e** The binding sites between miR-186 and SOX4 3′UTR predicted by StarBase. **f** The relationship between miR-186 and SOX4 verified by dual-luciferase reporter gene assay. **g**, **h** SOX4 mRNA (**g**) and protein (**h**) expression in the fibroblasts transfected with miR-186 mimic measured by RT-qPCR and western blot analysis. **i**, **j** The expression of miR-186 and SOX4 mRNA (**i**) and protein (**j**) expression measured by RT-qPCR and Western blot analysis after fibroblasts were transfected with miR-186 inhibitor and treated with BMSC-EVs. *p < 0.05 vs. the fibroblasts transfected with inhibitor-NC; ^#^*p* < 0.05 vs. the fibroblasts treated with miR-186 inhibitor and PBS. Differences between two groups were compared by unpaired *t* test, while differences among groups were determined by one-way ANOVA. The experiment was repeated three times

A total of 313 differentially expressed genes in PF were obtained from GEO database microarray GSE35145 (Fig. 4a), and 519 target genes of miR-186 were obtained from the StarBase databases. The above-mentioned differentially expressed genes and downstream target genes were intersected with human transcription factors obtained from Cistrome, the results of which demonstrated that SOX4 was the only key transcription factor with a significant different expression in PF (Fig. 4b). SOX4 has been previously reported to promote the occurrence of IPF [24]. The mRNA and protein expression of SOX4 was significantly upregulated in the lung tissues of IPF patients as compared to the control donors (Fig. 4c, d). Therefore, we further investigated whether miR-186 affected IPF by targeting SOX4. Binding sites between SOX4 and miR-186 were predicted both in human and mouse by StarBase (Fig. 4e).

Dual-luciferase reporter gene assay was performed to confirm the SOX4 and miR-186 binding relationship. The luciferase activity in cells co-transfected with miR-186 and wt-SOX4 3′UTR was significantly reduced, while that in cells co-transfected with miR-186 and mut-SOX4 3′UTR was unaltered (Fig. 4f). As shown in Fig. 4g, h, the expression of SOX4 was markedly downregulated in the fibroblasts transfected with miR-186 mimic compared to fibroblasts transfected with mimic-NC. To confirm the downregulation of SOX4 in the fibroblasts after EV treatment was indeed provoked by the miR-186 expression transferred to the fibroblasts, miR-186 was silenced in the fibroblasts which were subsequently treated with BMSC-EVs. Results showed that when miR-186 expression was significantly decreased in the fibroblasts, the expression of SOX4 was significantly upregulated. However, miR-186 expression was increased significantly upon treatment with BMSC-EVs, accompanied with the decreased expression of SOX4 (Fig. 4i, j).

### EV-miR-186 downregulates SOX4 expression to inhibit the expression of DKK1 and restrain fibroblast activation

**Fig. 5:**
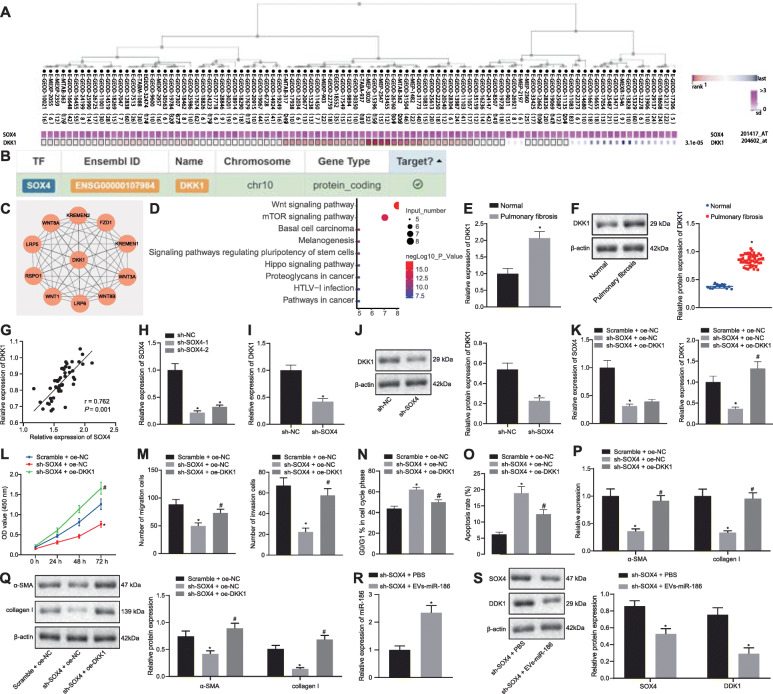
EV-delivered miR-186 decreases the expression of DKK1 through targeting SOX4 to impair fibroblast activation. **a** The significant co-expression relationship between SOX4 and DKK1 predicted by MEM (https://biit.cs.ut.ee/mem/index.cgi) (*p* = 3.1e−05). **b** The relationship between SOX4 and DKK1 predicted by hTFtarget (http://bioinfo.life.hust.edu.cn/hTFtarget#!/). **c** The PPI network of DKK1 and its related genes predicted by String (https://string-db.org) (the circle: the core degree of the gene = 10). **d** KEGG pathway map of DKK1 and the related genes analyzed by KOBAS (http://kobas.cbi.pku.edu.cn) (horizontal axis: enriched pathway; lateral axis: the number of genes enriched in this pathway; red indicates high significance; blue indicates low significance). **e**, **f** DKK1 mRNA (**e**) and protein (**f**) expression in lung tissues of patients with IPF (*n* = 48) and control donors (*n* = 16) measured by RT-qPCR and western blot analysis. **g** The correlation of DKK1 expression with SOX4 expression in clinical samples. **h** The silencing efficiency of sh-SOX4-1 and sh-SOX4-2 determined by RT-qPCR. **i**, **j** DKK1 mRNA (**i**) and protein (**j**) expression in fibroblasts after SOX4 silencing measured by RT-qPCR and Western blot analysis. **k** SOX4 and DKK1 mRNA expression in the fibroblasts after SOX4 knockdown/DKK1 overexpression determined by RT-qPCR. **l** The proliferation of LL29 fibroblasts after SOX4 knockdown/DKK1 overexpression analyzed by CCK8. **m** The migration and invasion of fibroblasts after SOX4 knockdown/DKK1 overexpression evaluated by Transwell assay. **n**, **o** Cell cycle (**n**) and apoptosis (**o**) after SOX4 knockdown/DKK1 overexpression determined by flow cytometry. **p**, **q** The mRNA (**p**) and protein (**q**) expression of α-SMA and collagen I in the fibroblasts after SOX4 knockdown/DKK1 overexpression measured by RT-qPCR and western blot analysis. **r** miR-186 expression in fibroblasts treated with EV-miR-186 after SOX4 knockdown determined by RT-qPCR. **s** The protein expression of SOX4 and DKK1 in fibroblasts treated with EV-miR-186 after SOX4 knockdown measured by Western blot analysis. In **k**–**p**, *p < 0.05 vs. the fibroblasts co-transfected with scramble and oe-NC; ^#^*p* < 0.05 vs. the fibroblasts co-transfected with sh-SOX4 and oe-NC. In **r** and **s**, *p < 0.05 vs. the fibroblasts treated with sh-SOX4, and PBS. Differences between two groups were compared by unpaired *t* test, while differences among groups were determined by one-way ANOVA. Besides, the comparison of the data in each group at different time points was analyzed by two-way ANOVA. Pearson correlation was used to analyze the correlation between SOX4 and DKK1. The experiment was repeated three times

A previous study has reported that SOX4 can regulate the expression of the downstream gene DKK1 [17]. Results from MEM prediction revealed a significant co-expression relationship between SOX4 and DKK1 (Fig. 5a). The hTFtarget prediction demonstrated that SOX4 could regulate DKK1 expression as a transcription factor (Fig. 5b), while String prediction revealed 10 genes related to DKK1 (Fig. 5c). The KEGG analysis demonstrated that DKK1 and the aforementioned 10 genes were mainly engaged in the Wnt signaling pathway, mTOR signaling pathway, and basal cell carcinoma (Fig. 5d). Notably, previous studies showed that Wnt and mTOR signaling pathways are associated with PF [25, 26]. Hence, we assumed that miR-186 affects PF development through SOX4-mediated DKK1 expression. Next, the expression of DKK1 in clinical sample tissues was determined by RT-qPCR and Western blot analysis, and the correlation between the expression of DKK1 and SOX4 in PF was analyzed. As shown in Fig. 5e–g, the expression of DKK1 was appreciably upregulated in the lung tissues of patients with IPF as compared to control donors and DKK1 expression was positively correlated with SOX4 expression. Therefore, we further investigated whether the knockdown of SOX4 changed DKK1 expression. First, we designed two shRNAs targeting SOX4 and determined the transfection efficiency upon sh-SOX4 transfection into the fibroblasts (Fig. 5h). The results showed that both sh-SOX4-1 and sh-SOX4-2 could successfully reduce the expression of SOX4 in fibroblasts. We hence used sh-SOX4-1 with higher silencing efficiency. As a consequence, DKK1 expression was decreased by SOX4 knockdown in fibroblasts (Fig. 5i–j). In addition, DKK1 was overexpressed simultaneously in the SOX4-deficient fibroblasts. The expression of SOX4 in SOX4-deficient fibroblasts was unaffected while expression of DKK1 was rescued by oe-DKK1 transfection (Fig. 5k). To explore the effect of SOX4/DKK1 on fibroblast activation, we examined cell proliferation, migration and invasion, cell cycle and apoptosis, and the expression of myofibroblastic markers α-SMA and collagen I. The proliferative, migrating, and invasive properties of fibroblasts were suppressed upon SOX4 knockdown, all of which were rescued by restoration of DKK1 expression (Fig. 5l, m). The proportion of fibroblasts in G0/G1 phase and apoptosis were increased by SOX4 silencing, whereas these changes were reversed by oe-DKK1 (Fig. 5n, o). Meanwhile, expression of α-SMA and collagen I was prominently decreased in the SOX4-deficient fibroblasts but was increased by oe-DKK1 transfection (Fig. 5p, q). These results confirmed that SOX4 promoted the expression of DKK1 and resulted in fibroblast activation. To determine further whether EV-miR-186 affected SOX4-dependent upregulation of DKK1, the SOX4-deficient fibroblasts were treated with EV-miR-186. As shown in Fig. 5r, s, the expression of SOX4 and DKK1 was downregulated by EV-miR-186 treatment in SOX4-deficient fibroblasts, which indicated that EV-miR-186 could reduce DKK1 expression by downregulating SOX4 expression. Based on these findings, we concluded that EV-miR-186 could reduce the expression of DKK1 through the downregulation of SOX4, thereby disrupting the activation of fibroblasts.

### EV-miR-186 alleviates PF in vivo

**Fig. 6:**
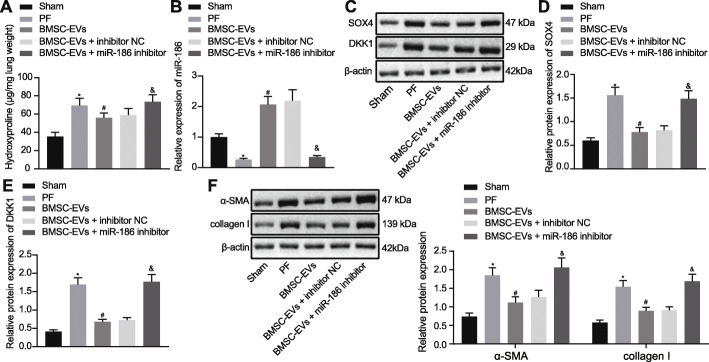
EV-miR-186 alleviates PF in vivo. **a** Hydroxyproline content in the lung tissues of mice. **b** miR-186 expression in the lung tissues of mice determined by RT-qPCR. **c**–**e** The protein expression of SOX4 and DKK1 in the lung tissues of mice determined by Western blot analysis. **f** The protein expression of α-SMA and collagen I in the lung tissues of mice measured by Western blot analysis. *p < 0.05 vs. the sham-operated mice; ^#^*p* < 0.05 vs. the mice with PF; ^&^*p* < 0.05 vs. the mice with PF treated with EVs from inhibitor NC-transfected BMSCs. Differences among groups were determined by one-way ANOVA, *N* = 8

We investigated further the effect of miR-186 in BMSC-EVs on PF in vivo. Results from HE staining demonstrated that BMSC-EVs treatment partially alleviated the PF in the mice. The lung tissue of mice showed significant PF pathology when miR-186 expression in BMSC-EVs was suppressed (Supplementary Figure 1A). A similar result was obtained by Masson staining (Supplementary Figure 1B), which identified that the collagen fibers in mice with PF were reduced after treatment with BMSC-EVs. However, the collagen fibers remained abundant when miR-186 expression in BMSC-EVs was suppressed. In addition, significant upregulation of collagen in the mice with PF was observed by Sirius Red staining, which was partially reduced after treatment with BMSC-EVs. Interestingly, when the miR-186 expression in BMSC-EVs was suppressed, an upregulation of collagen was detected (Supplementary Figure 1C). Additionally, we assessed the effects of BMSC-EVs on the number of fibroblasts by immunofluorescence. The results revealed an increased number of fibroblasts in the mice with PF, as shown by enhanced fluorescence intensity in the mouse lung tissues. However, the treatment with BMSC-EVs reduced the number of fibroblasts in the lung tissues of mice with PF. When the miR-186 expression in BMSC-EVs was suppressed, the number of fibroblasts was partially rescued (Supplementary Figure 1D). The hydroxyproline content was increased in the lung tissues of the mice with PF as compared to the sham-operated mice, and this increase was counteracted by BMSC-EVs. Also, hydroxyproline content in the lung tissues of the mice with PF was increased when miR-186 expression in BMSC-EVs was suppressed (Fig. 6a). Next, we measured the expression profiles of miR-186 (Fig. 6b), SOX4, and DKK1 (Fig. 6c, d) in lung tissues. The results showed that, compared to the sham-operated mice, miR-186 was downregulated while SOX4 and DKK1 was upregulated in mice with PF. After treatment with BMSC-EVs in the mice with PF, the expression of miR-186 was notably increased while the SOX4 and DKK1 expression was markedly decreased. In comparison with the treatment of EVs isolated from inhibitor NC-transfected BMSCs, treatment with EVs isolated from miR-186 inhibitor-transfected BMSCs led to upregulated expression of SOX4 and DKK1 and downregulated expression of miR-186, thus indicating that miR-186 in BMSC-EVs targeted and inhibited SOX4 expression, thereby abolishing the expression of DKK1. In addition, we detected the expression of α-SMA and collagen I upon BMSC-EV treatment. As shown in Fig. 6e, f, the treatment with BMSC-EVs resulted in decreased expression of α-SMA and collagen I, which was increased after miR-186 expression in BMSC-EVs was suppressed. These results further demonstrated that EV-miR-186 could target SOX4 and downregulate DKK1 to impede the occurrence of IPF.

## Discussion

Idiopathic pulmonary fibrosis (IPF) is a progressive lung disease often characterized by subpleural fibrosis, subepithelial fibroblast foci, and microscopic honeycombing [27]. It has been reported that two thirds of patients with IPF survive 2 to 5 years after diagnosis [28]. The key reason of short survival rate is that the fibrosis spreads into contiguous alveoli, establishing a reticular network that leads to fatal asphyxiation [29]. The regulatory role of BMSC-EVs carrying miR-186 in the progression of IPF was investigated in our study. Our results demonstrated that miR-186 carried by BMSC-EVs suppressed the proliferation, invasion, and differentiation of the fibroblasts by downregulating SOX4 and DKK1, thus delaying the development of PF pathology.

Initially, we identified that BMSC-EVs suppressed fibroblast proliferation and promoted their apoptosis, hence inhibiting the activation of fibroblasts. Meanwhile, BMSC-EVs ameliorated PF in vivo by reducing the collagen overexpression and hydroxyproline content in the affected lungs. A previous study has reported that MSCs exert a protective effect on pulmonary function in IPF [30], which was in line with our study results. The hepatoprotective effects of MSCs have been demonstrated in hepatic ischemia-reperfusion injury to be mediated by releasing EVs [31]. Besides, treatment of BMSC-EVs in the mice with acute graft-versus-host disease has been shown to alleviate the damage to the targeted organs [32]. Of note, EVs can deliver biomolecules such as proteins, mRNA, microRNA, and DNA during cell-to-cell communication and thus affect the microenvironment of the lungs [33]. Therefore, we further analyzed the mechanism by which EVs functioned to ameliorate PF.

Subsequently, we identified that miR-186 was poorly expressed in the tissues of patients with IPF, but that expression was enriched in BMSC-EVs. Our data revealed that inhibition of miR-186 reversed the effect of BMSC-EVs on the activation of the fibroblasts. Hence, it is reasonable to conclude that BMSC-EVs can deliver a cargo of miR-186 to repress fibroblast activation. In fact, there were several miRNAs that have been reported to be involved in the pathogenesis of IPF [34]. For instance, the typically poorly expressed miR-338 (miR-338-5p) has been identified in the fibroblasts and lung fibrotic tissues induced by TGF-β treatment, while the upregulated miR-338 expression has been shown to suppress the progression of PF [35]. Another miRNA, miR-26a, mediates the Lin28B/let-7d axis to exacerbate the epithelial-mesenchymal transition in IPF [36]. Also, miR-497 has been showed to hinder the excessive proliferation of lung fibroblasts induced by TGFβ1 [37]. Consistent with our present findings, miR-186 can disrupt collagen V overexpression during IPF [13]. More recently, miR-186 has been shown to impair the proliferation of inflammatory fibroblasts through regulation of HIF-1α in chronic obstructive pulmonary disease [14]. Our in vivo experiments further demonstrated that miR-186 released from BMSC-EVs could reduce collagen and hydroxyproline contents and downregulate α-SMA and collagen I, suggesting its protective effect against IPF.

Most importantly, we found that SOX4 was a target gene of miR-186 and that SOX4 was an upregulated gene in PF. In a preceding study, SOX4 upregulation was suggested to be associated with the progression of IPF [38]. Known as a fibrogenic gene, SOX4 potentially contributes to activation of hepatic stellate cells and promotion of PF pathology [39]. Furthermore, the interaction between miR-186 and SOX4 and their functional mechanisms in IPF have been addressed in our study. A similar mechanism was also found in another study, whereby miR-129-5p targeted SOX4 3′UTR and suppressed the fibrosis occurring due to peritoneal dialysis [40].

## Conclusion

The key finding was the demonstration of a mitigatory role of miR-186 in BMSC-EVs via interaction with SOX4 and DKK1 in PF. The BMSC-EVs successfully delivered miR-186 into fibroblasts and inhibited their activation by suppressing SOX4 and DKK1, thereby alleviating PF. These findings suggest that miR-186 carried by BMSC-EVs may serve as a promising therapeutic target in treating human IPF. We believe that the BMSC-EV therapies may provide new means and assistance for the treatment of IPF. At present, we contend that there is an urgent need to proceed to clinical trials employing the present strategy and doses of BMSC-EVs for targeted delivered delivery of miR-186 to patients with IPF to test the feasibility and safety in clinical applications.

## Supplementary Information

**Additional file 1: Supplementary Figure 1**. EVs-miR-186 alleviates PF in vivo*.* A, HE staining of the lung tissues in mice with PF (200 ×). B, Masson staining of the lung tissues in mice with PF (200 ×).C, Sirius Red staining of the lung tissues in mice with PF (200 ×). D, Immunofluorescence for the number of fibroblasts in the lung tissues in mice with PF, where red fluorescence refers to TE-7 antibdody staining and blue fluorescence refers to DAPI staining.

## Data Availability

Data sharing not applicable to this article as no datasets were generated or analyzed during the current study.

## Abbreviations

IPF: Idiopathic pulmonary fibrosis
BMSC-EVs: Bone marrow mesenchymal stem cell-derived extracellular vesicles
CCK-8: Cell Counting Kit-8
SOX4: SRY-related HMG box transcription factor 4
DKK1: Dickkopf-1
MSCs: Mesenchymal stem cells
GEO: Gene Expression Omnibus
KOBAS: KO-Based Annotation System
MRI: Magnetic resonance imaging
FBS: Fetal bovine serum
